# Peutz-Jeghers Syndrome With Incidental Legg-Calvé-Perthes Disease: A Rare Radiological Association in a Pediatric Patient

**DOI:** 10.7759/cureus.110384

**Published:** 2026-06-07

**Authors:** Vadakattu Mani Harsha, Ravi Kiran, Abburi Anil Kumar, Rupesh M, Prasanthi Gunnu

**Affiliations:** 1 Radiodiagnosis, Great Eastern Medical School and Hospital, Srikakulam, IND

**Keywords:** computed tomography, hamartomatous polyps, incidental findings, intussusception, legg-calvé-perthes disease, pediatric radiology, peutz-jeghers syndrome

## Abstract

Peutz-Jeghers syndrome (PJS) is a rare autosomal dominant hamartomatous polyposis disorder characterised by mucocutaneous pigmentation and multiple gastrointestinal polyps, most commonly involving the small intestine. Paediatric patients frequently present with complications, such as abdominal pain, gastrointestinal bleeding, and recurrent intussusception. We report the case of a nine-year-old female with classical clinical and radiological features of PJS, including multisite gastrointestinal polyposis and a prior history of intussusception. Contrast-enhanced computed tomography revealed multiple heterogeneously enhancing sessile polyps involving the stomach, duodenum, colon, and rectum. Histopathological examination confirmed the diagnosis of PJS. In addition, imaging demonstrated incidental findings of femoral head epiphyseal flattening and sclerosis, consistent with avascular necrosis, suggestive of Legg-Calvé-Perthes disease (LCPD). A comprehensive review of the available literature revealed no previously reported cases describing a similar radiological association between PJS and LCPD. While both conditions are individually well documented, their coexistence appears to be extremely rare, thereby highlighting the novelty of the present case. The patient was managed conservatively and is currently under follow-up, with a stable clinical status. This case underscores the value of comprehensive cross-sectional imaging in pediatric patients with syndromic disorders, facilitating both diagnostic confirmation and the identification of unexpected but clinically significant incidental findings.

## Introduction

Peutz-Jeghers syndrome (PJS) is a rare autosomal dominant hamartomatous polyposis disorder characterized by distinctive mucocutaneous pigmentation and multiple gastrointestinal polyps, most commonly involving the small intestine [1,2]. The condition is associated with mutations in the STK11/LKB1 tumor suppressor gene; however, in many clinical settings, the diagnosis is established based on characteristic phenotypic features and clinical criteria when genetic testing is unavailable [3]. Pediatric patients with PJS frequently present with abdominal pain, gastrointestinal bleeding, anemia, or complications such as bowel obstruction and recurrent intussusception, often necessitating repeated imaging and surgical interventions. Cross-sectional imaging plays a pivotal role in evaluating polyp burden, detecting complications, and guiding long-term surveillance. Legg-Calvé-Perthes disease (LCPD) is an idiopathic form of avascular necrosis of the femoral head occurring predominantly in children between four and ten years of age [4]. The underlying pathophysiology is believed to involve transient disruption of the epiphyseal blood supply, with multifactorial contributions, including vascular abnormalities, mechanical factors, and thrombophilic tendencies [4]. Clinical presentation may be subtle, with limping or limited hip movement, and pain may be minimal or absent, leading to delayed diagnosis. Affected children carry a higher likelihood of future hip osteoarthritis [5]. Radiological imaging remains central to the detection and staging of the disease, particularly in early or asymptomatic cases. Musculoskeletal involvement is not a recognized feature of PJS, and no established association between PJS and avascular necrosis of the femoral head has been described. A comprehensive literature search was performed in PubMed, Google Scholar, and Scopus using keywords including PJS, LCPD, and coexistence. However, no prior reports describing their coexistence were identified. In this report, we describe a pediatric patient with classical clinical and radiological features of PJS in whom contrast-enhanced computed tomography incidentally revealed imaging findings consistent with LCPD. This case highlights the importance of systematic evaluation of all anatomical regions on cross-sectional imaging in syndromic children, as incidental findings may have significant implications for patient management and follow-up.

## Case presentation

A nine-year-old female child presented with a history of intermittent abdominal pain and episodic vomiting. The patient had a significant history of intussusception, for which she underwent surgical management two years prior. There was no documented family history of gastrointestinal polyposis syndromes. On clinical examination, the child was hemodynamically stable. Physical examination revealed characteristic mucocutaneous hyperpigmentation involving the upper and lower lips and perioral region (Figure 1). No additional cutaneous abnormalities were noted. Abdominal examination at presentation was unremarkable, with no palpable mass or clinical features suggestive of acute intestinal obstruction.

**Figure 1:**
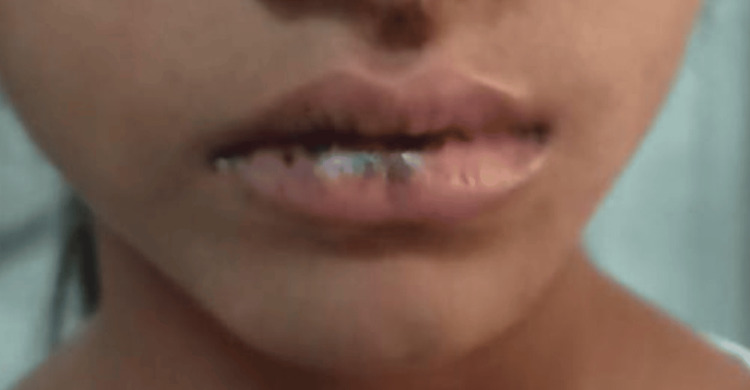
Multiple hyperpigmented macules located on the lips.

Complete blood count parameters, including red blood cell count, white blood cell count, hemoglobin, hematocrit, and platelet count, were within normal reference ranges (Table 1). In view of the recurrent abdominal symptoms and prior surgical history, Contrast-enhanced computed tomography (CECT) of the abdomen was performed using a 16-slice GE multidetector computed tomography scanner. Intravenous access was secured with a 22-gauge peripheral cannula in the dorsal hand vein. A non-ionic iodinated contrast agent was administered intravenously at a dose of 1.5-2 mL/kg using an automated power injector at a flow rate of 2-3 mL/s, followed by a saline flush. Images were acquired in the arterial, porto-venous, venous, five minutes delayed phases. The child tolerated the procedure well, with no immediate contrast-related adverse events. Contrast-enhanced computed tomography demonstrated multiple sessile, heterogeneously enhancing intraluminal lesions consistent with gastrointestinal polyps (Figure 2). A well-defined sessile polyp measuring approximately 1.9 × 1.4 cm was identified arising from the lesser curvature of the stomach, located approximately 4 cm from the gastroesophageal junction (Figure 2A). Additionally, polypoidal lesions were noted in the second part of the duodenum (Figure 2B). In the large bowel, multiple enhancing polypoidal lesions were identified in the rectum (Figure 2C). A larger sessile polyp measuring approximately 3.0 × 2.2 cm was noted in the sigmoid colon (Figure 2D). No evidence of acute intussusception, bowel ischemia, or perforation was identified at the time of imaging. Upper gastrointestinal endoscopy and colonoscopy were performed as part of the gastrointestinal evaluation, which demonstrated multiple gastrointestinal polyps.

**Table 1:** Complete blood count parameters and reference ranges. WBC: white blood cells; RBC: red blood cells

Variables	Patient values	Normal range
WBC	10.11	4.0-11.0 x 10^3^/uL
RBC	4.81	4.5-6.0 x 10^6^/uL
Hemoglobin	12.6	11.0-16.0 gm/dL
Hematocrit	39.8%	35.0-45.0%
Platelet count	352	150-400 x 10^3^/uL

**Figure 2:**
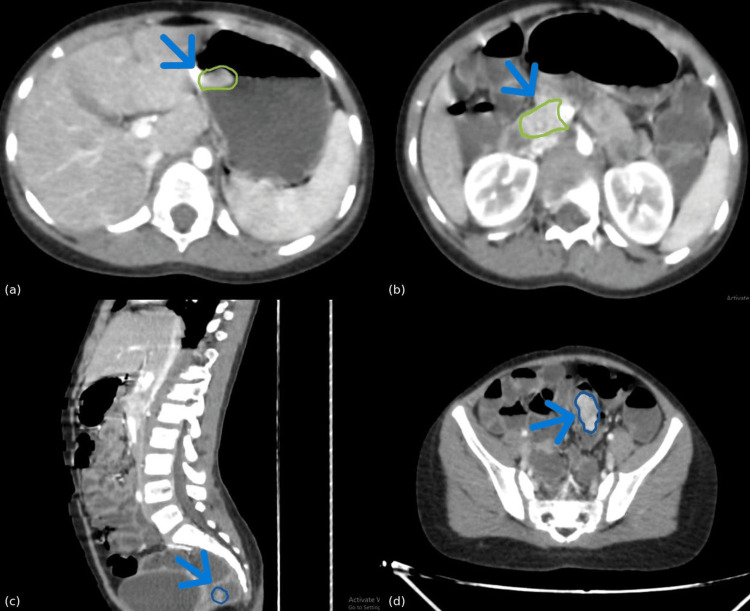
Contrast-enhanced computed tomography (CECT) of the abdomen demonstrating multisite gastrointestinal polyps. (A) Axial image showing a heterogeneously enhancing sessile polyp arising from the lesser curvature of the stomach (arrow). (B) Axial image demonstrating an enhancing intraluminal polyp within the second part of the duodenum (arrow). (C) Axial pelvic image showing multiple enhancing polypoidal lesions involving the rectum (arrows). (D) Axial pelvic image demonstrating a well-defined, heterogeneously enhancing sessile polyp within the sigmoid colon (arrow).

Incidentally, evaluation of the bone window images revealed mild flattening with focal fragmentation of the right femoral head epiphysis, accompanied by surrounding sclerosis. This was noted as an incidental finding. These imaging features were consistent with avascular necrosis of the femoral head, suggestive of LCPD (Figure 3). On further clinical evaluation, the child had a normal gait with no limping. Hip examination revealed no restriction of movement, and the musculoskeletal findings were clinically asymptomatic at presentation.

**Figure 3:**
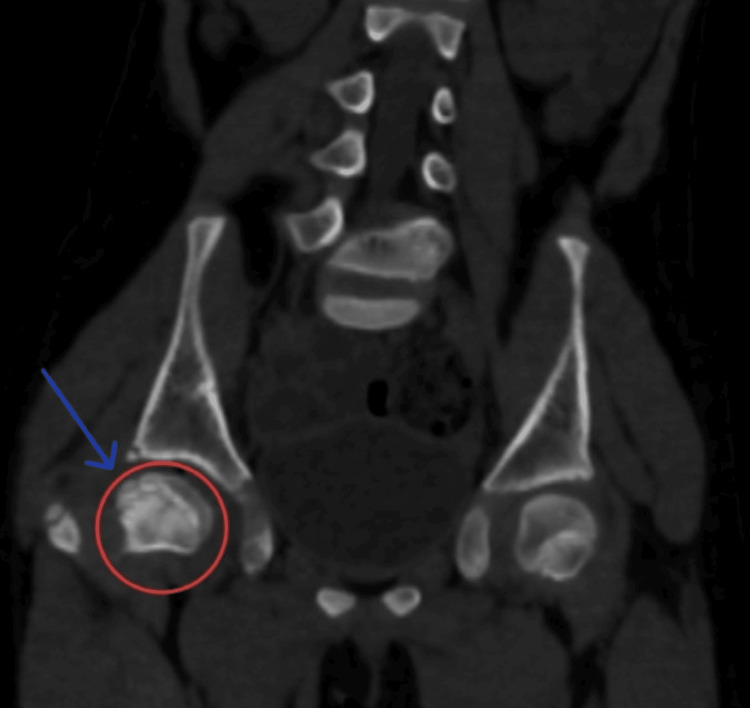
Imaging showing mild flattening and focal fragmentation of the right femoral head epiphysis (blue arrow) with areas of sclerosis, suggestive of avascular necrosis of the femoral epiphysis (Legg-Calvé-Perthes disease).

Based on the presence of characteristic mucocutaneous pigmentation, multisite gastrointestinal hamartomatous polyps, and a prior history of intussusception, a diagnosis of PJS was initially established on clinical and radiological grounds. The incidental imaging finding suggestive of LCPD was documented, and the patient was initially referred for orthopedic evaluation and follow-up. Subsequently, owing to symptomatic gastrointestinal polyps, the patient underwent segmental bowel resection. Histopathological examination of the resected specimen confirmed the diagnosis, demonstrating features consistent with a Peutz-Jeghers hamartomatous polyp. The patient was advised to continue gastroenterology surveillance for long-term monitoring and management. Conservative management, including activity modification and regular orthopedic follow-up, was advised with emphasis on preserving hip joint mobility and monitoring for disease progression. As the lesion was incidentally detected on contrast-enhanced CT imaging and the patient remained asymptomatic, additional magnetic resonance imaging was not considered necessary at the time.

## Discussion

This case highlights a rare and previously unreported coexistence of clinically diagnosed PJS with histopathologic confirmation of hamartomatous polyps and incidental radiological imaging findings suggestive of LCPD in a pediatric patient. A comprehensive review of the available literature revealed no previously reported cases describing a similar radiological association. While the coexistence is most likely incidental, its recognition is important from a radiological and clinical perspective, particularly in children undergoing cross-sectional imaging for gastrointestinal indications. PJS is a hereditary hamartomatous polyposis disorder characterized by mucocutaneous hyperpigmentation and multiple gastrointestinal polyps, predominantly involving the small intestine, followed by the colon and stomach [1]. Pediatric patients frequently present with abdominal pain, gastrointestinal bleeding, anemia, or complications such as bowel obstruction and intussusception. Intussusception remains one of the most common acute presentations in children with PJS and often necessitates surgical intervention early in life.

Although mutations in the STK11/LKB1 gene represent the molecular hallmark of PJS, many patients continue to be diagnosed clinically using established diagnostic criteria, particularly in settings where genetic testing is not readily available [3]. In the present case, the diagnosis of PJS was initially established on clinical and radiological grounds, based on characteristic mucocutaneous pigmentation, multisite gastrointestinal polyposis, and a prior history of intussusception, and was subsequently confirmed by histopathological examination of the resected bowel specimen, which demonstrated features consistent with a Peutz-Jeghers hamartomatous polyp. Originally described by Peutz in 1921 and later by Jeghers in 1944, the syndrome is defined by characteristic mucocutaneous pigmentation, present in approximately 95% of cases, and multiple gastrointestinal hamartomatous polyps [6,7]. In patients with PJS, hamartomatous polyps most frequently involve the jejunum, followed by the ileum and duodenum [8]. Cross-sectional imaging plays a central role in the evaluation and surveillance of patients with PJS. Contrast-enhanced computed tomography is particularly useful for assessing polyp burden, detecting complications such as obstruction or intussusception, and guiding management decisions. Importantly, computed tomography imaging also allows evaluation beyond the gastrointestinal tract, facilitating detection of incidental but clinically relevant findings. In this patient, computed tomography imaging not only demonstrated multiple gastrointestinal polyps involving the stomach, duodenum, colon, and rectum but also revealed osseous changes involving the femoral head.

LCPD is a recognized cause of avascular necrosis of the femoral head in childhood and is thought to arise from temporary disruption of the epiphyseal blood supply [4]. Owing to its often subtle or absent clinical manifestations, particularly during later or reparative phases, the disease may remain clinically silent and be identified incidentally on imaging performed for unrelated indications [9]. In the present case, computed tomography demonstrated femoral head epiphyseal flattening with associated sclerosis, findings consistent with LCPD. The lack of hip pain, gait abnormality, or functional limitation further supports an asymptomatic clinical course. Musculoskeletal abnormalities are not recognised features of the phenotypic spectrum of PJS. Although the STK11/LKB1 signaling pathway has roles in cellular metabolism, angiogenesis, and endothelial regulation, current clinical evidence does not support a relationship between PJS and avascular necrosis, thrombophilia, or coagulation disorders. While vascular compromise is implicated in the pathogenesis of LCPD, no published data establish a link between this mechanism and PJS. Genetic testing for STK11/LKB1 mutation analysis was not undertaken in the present case due to the unavailability in our setting. Accordingly, the concurrence of these two conditions in the present patient is most appropriately interpreted as incidental, and a causal association should not be inferred. This case underscores the importance of comprehensive and systematic evaluation of cross-sectional imaging in pediatric patients with syndromic disorders. Incidental findings, even when unrelated to the primary diagnosis, may have important implications for patient counseling, multidisciplinary referral, and long-term follow-up. Early recognition of conditions such as LCPD enables timely orthopedic assessment and appropriate surveillance, ultimately contributing to improved patient care.

## Conclusions

The incidental identification of imaging features consistent with LCPD in a pediatric patient with clinically diagnosed PJS with histopathological confirmation of hamartomatous polyps represents a rare and, to our knowledge, previously unreported coexistence. Although this association is most likely incidental, it emphasizes the importance of systematic evaluation of all visualized structures on cross-sectional imaging performed for syndromic surveillance. Comprehensive imaging assessment not only aids in confirming the primary diagnosis but also facilitates early recognition of unrelated conditions with potential long-term implications. Documentation of such incidental findings may contribute to a broader understanding of syndromic presentations and support multidisciplinary follow-up and patient-centered care.
